# Interoperability of heterogeneous health information systems: a systematic literature review

**DOI:** 10.1186/s12911-023-02115-5

**Published:** 2023-01-24

**Authors:** Amir Torab-Miandoab, Taha Samad-Soltani, Ahmadreza Jodati, Peyman Rezaei-Hachesu

**Affiliations:** 1 Department of Health Information Technology, Faculty of Management and Medical Informatics, Tabriz University of Medical Sciences, Golghast St., Tabriz, 5166614711 Iran; 2 Cardiovascular Research Center, Tabriz University of Medical Sciences, Tabriz, Iran

**Keywords:** Electronic health record, Health information systems, Heterogeneous, Interoperability, Requirements

## Abstract

**Background:**

The lack of interoperability between health information systems reduces the quality of care provided to patients and wastes resources. Accordingly, there is an urgent need to develop integration mechanisms among the various health information systems. The aim of this review was to investigate the interoperability requirements for heterogeneous health information systems and to summarize and present them.

**Methods:**

In accordance with the PRISMA guideline, a broad electronic search of all literature was conducted on the topic through six databases, including PubMed, Web of science, Scopus, MEDLINE, Cochrane Library and Embase to 25 July 2022. The inclusion criteria were to select English-written articles available in full text with the closest objectives. 36 articles were selected for further analysis.

**Results:**

Interoperability has been raised in the field of health information systems from 2003 and now it is one of the topics of interest to researchers. The projects done in this field are mostly in the national scope and to achieve the electronic health record. HL7 FHIR, CDA, HIPAA and SNOMED-CT, SOA, RIM, XML, API, JAVA and SQL are among the most important requirements for implementing interoperability. In order to guarantee the concept of data exchange, semantic interaction is the best choice because the systems can recognize and process semantically similar information homogeneously.

**Conclusions:**

The health industry has become more complex and has new needs. Interoperability meets this needs by communicating between the output and input of processor systems and making easier to access the data in the required formats.

**Supplementary Information:**

The online version contains supplementary material available at 10.1186/s12911-023-02115-5.

## Background

Every society requires quality and reliable healthcare, which is employed as a structural part of daily life. Because of new diagnostic and therapeutic ways and means, new procedures, and the existence of multiple professional groups, each with its own set of characteristics, requirements, and working methods, the healthcare field is exceedingly complicated [1]. As a result, the use of health information technologies is required to ensure the long-term stability of the healthcare system. In this regard, various health information technologies have been developed for the translation of paper-based health information into electronic health information throughout the last few decades, and many hospitals around the world have implemented health information systems [2].

A health information system is broadly defined as a system that integrates data collection, processing, reporting, and use of the information necessary for improving health service effectiveness and efficiency through better management at all levels of health services [3, 4]. These systems, contribute to a better coordination of care, better organization of information, timeliness, accuracy and completeness of information, the ability to analyze information, reduce medical errors, reduce costs, continuity of care, information exchange, quick and easy access to providers and information in different places and times, and the improvement of the communication between health professionals and patients [5].

The benefits of health institutions adopting health information systems are apparent. However, the systems currently in use are proprietary, may differ from one health institution to the next, and were developed for local access, resulting in heterogeneity in the existing ecosystems [6]. The health information of a patient may be distributed among an unspecified number of healthcare facilities. A hospital's medical information are not interchangeable and accessible to another health institution. In a given situation, the health professional does not have access to the whole of the patient's medical information unless the patient is conscious and can provide the information that the health professional requires to make an informed and individualized decision on the best course of action at the time [7]. To make an informed decision on which procedures to follow, a healthcare professional needs access to information distributed across various institutions. If an institution makes an error due to a lack of information, overcoming the problem will be more difficult, if not impossible. Accordingly, there is an urgent need to develop integration mechanisms among the various health information systems to allow for ubiquitous access to patient health information [8].

The sharing of information among different levels of healthcare has a link to the quality, efficiency, and safety of care provided to a patient. The ability of systems to connect and exchange information with each other, in either implementation or access, without limitation refers to interoperability [9]. The interoperability between health information systems is of utmost importance and essential for a better health service management, public health, quality and safety of care to patients and clinical research. The lack of interoperability leads to redundant, disorganized, disjointed and inaccessible medical information, that may affect the quality of care provided to patients and waste of financial resources [10].

Despite the importance of the interoperability of health information systems, the various approaches taken to create it, and the growing academic interest in it, this issue is currently in a state of disarray. To the best of our knowledge, there has been no systematic study of the issue of interoperability of health information systems. Therefore, in the context of interoperability of health information systems, more emphasis is needed. This creates a need for collecting a dataset that consists of several research articles in the field of interoperability of health information systems from different scientific databases, and applying the proposed approach on them. The purpose of this review was to investigate the interoperability requirements for heterogeneous health information systems and to summarize and present them.

## Materials and methods

This review followed the preferred reporting items for systematic reviews and meta-analyses (PRISMA) guidelines for authors reporting a systematic review. PRISMA is an evidence-based minimum set of items for reporting in systematic reviews and meta-analyses. PRISMA primarily focuses on the reporting of reviews evaluating the effects of interventions, but can also be used as a basis for reporting systematic reviews with objectives other than evaluating interventions (e.g. evaluating etiology, prevalence, diagnosis or prognosis). PRISMA aims to help authors improve the reporting of systematic reviews and meta-analyses. PRISMA may also be useful for critical appraisal of published systematic reviews, although it is not a quality assessment instrument to gauge the quality of a systematic review [11].

### Information source

Studies were identified to 25 July 2022. They were selected by searching the online databases PubMed, Web of science, Scopus, MEDLINE, Cochrane Library and Embase, and Search Google Scholar. Searches included online books, published papers, conference abstracts and seminar and reference publications to avoid bias in publishing and to ensure that as many articles as possible were included. Additionally, the selected articles reference lists were searched for other relevant studies. In addition, the bibliographies of articles and reviews published were searched by hand for potentially relevant articles. An email alert function in the electronic databases was created to keep track of any newly released publications that meet the selection criteria based on the saved search history by 25 July 2022.

### Eligibility criteria

Two reviewers separately assessed the titles of the articles. The articles were classified into a "definitely exclude" category and a "possibly include" category; the abstract of any of the possibly include articles were assessed. Studies that contained abstracts that did not meet the criteria for inclusion were excluded. The entire text of the remaining articles was assessed and duplicate articles were deleted. Articles were eligible when they reported development, validation or translation studies of health information systems in the context of interoperability. In addition, only articles written in English and of which access to full text was available were included. Letters to the editor, commentary, review and opinion papers were also excluded. There was no limit on publishing year.

### Search

The search terms were derived from the concepts in the research objective: Electronic Medical Record, Electronic Health Record, Computerized Medical Record, Automated Medical Record, Hospital Information System, Health Information System, Clinical Information System, Medical Record System and Interoperability. This resulted in the following syntax.

("Electronic Medical Record" OR EMR OR "Electronic Health Record" OR EHR OR "Computerized Medical Record" OR CMR OR "Automated Medical Record" OR AMR OR "Hospital Information System" OR "Health Information System" OR HIS OR "Clinical Information System" OR CIS OR "Medical Record System") AND Interoperability

Potentially relevant articles were selected first based on title and imported into the Endnote. Further selection was performed on the basis of the articles' abstract and full text.

### Article selection

**Fig. 1:**
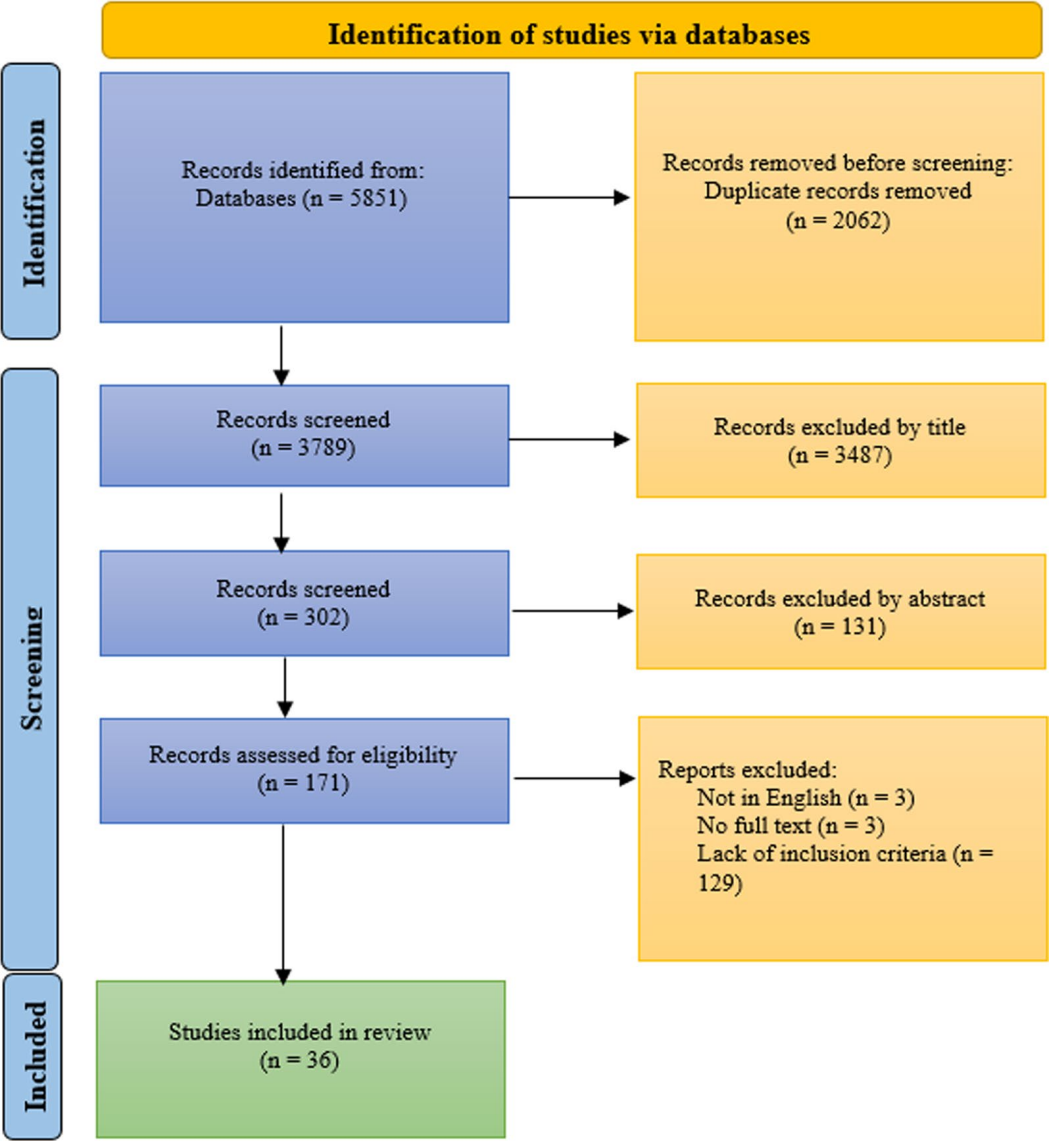
PRISMA flow diagram

An overview of the whole article selection process is shown in the PRISMA flow chart in Fig. 1. Three hundred and two articles based on title were selected. The abstracts were analyzed and 131 articles were excluded as they did not meet the eligibility criteria. The remaining 171 articles were further assessed for eligibility by reading the full text. 135 were excluded from this. Finally, 36 articles were included in the analysis.

### Data collection process

The full text of the chosen articles was then independently reviewed by the reviewer. The standardized data extraction form was used to extract the data from the selected articles. The data extraction sheet for each study included the authors, year, country, aim of research, interoperable systems, architecture and components, setting, included processes, used standards, platform and technology, extent of implementation, level of interoperability, information resource and key findings. All results were collated from the selected studies. Another independent investigator reviewed and verified for completeness and accuracy of all data entries.

### Quality assessment

The quality of the initially chosen studies was assessed and analyzed to support the inclusion / exclusion process. The used critical appraisal tool was a quality assessment of diagnostic accuracy studies (QUADAS) checklist. This tool was designed for testing combined reliability and validity studies, or for testing validity and reliability as separate components [12]. The checklist contains 14 questions with 3 options: yes, no and unclear. Two researchers analyzed all of the identified studies for risk of bias. In this way, if the information in each checklist question was provided in the study, this item was scored as "yes". If the information in each checklist question was not clearly reported in the study, this item was scored as "no", and in the case of incomplete information being reported, this item was scored as "unclear". Based on past studies, a score of 7 of 14 or greater of “yes's” indicates high quality study and scores below 7 indicate low quality study. Studies were considered to be of high quality provided. All differences were overcome by agreement. In order to present the results, Excel, XMind, online word cloud generator and yED graph editor software were used.

## Results

Figure 1 presents a flow diagram based on the PRISMA guidelines, which details the movement of articles through the review process. In total, 5851 titles were retrieved from the databases and 3789 titles remained after duplicated articles were removed. Two reviewers screened the titles and abstracts for relevance, and 3487 articles were deemed irrelevant to the review topic and were excluded. Three hundred and two articles were selected based on title. The abstracts were analyzed and 131 articles were excluded as they did not meet the eligibility criteria. In total, 171 full-text articles were retrieved and assessed according to the eligibility criteria. After the assessment, 135 articles were excluded (refer to Fig. 1) and 36 articles were included in the review.

**Fig. 2:**
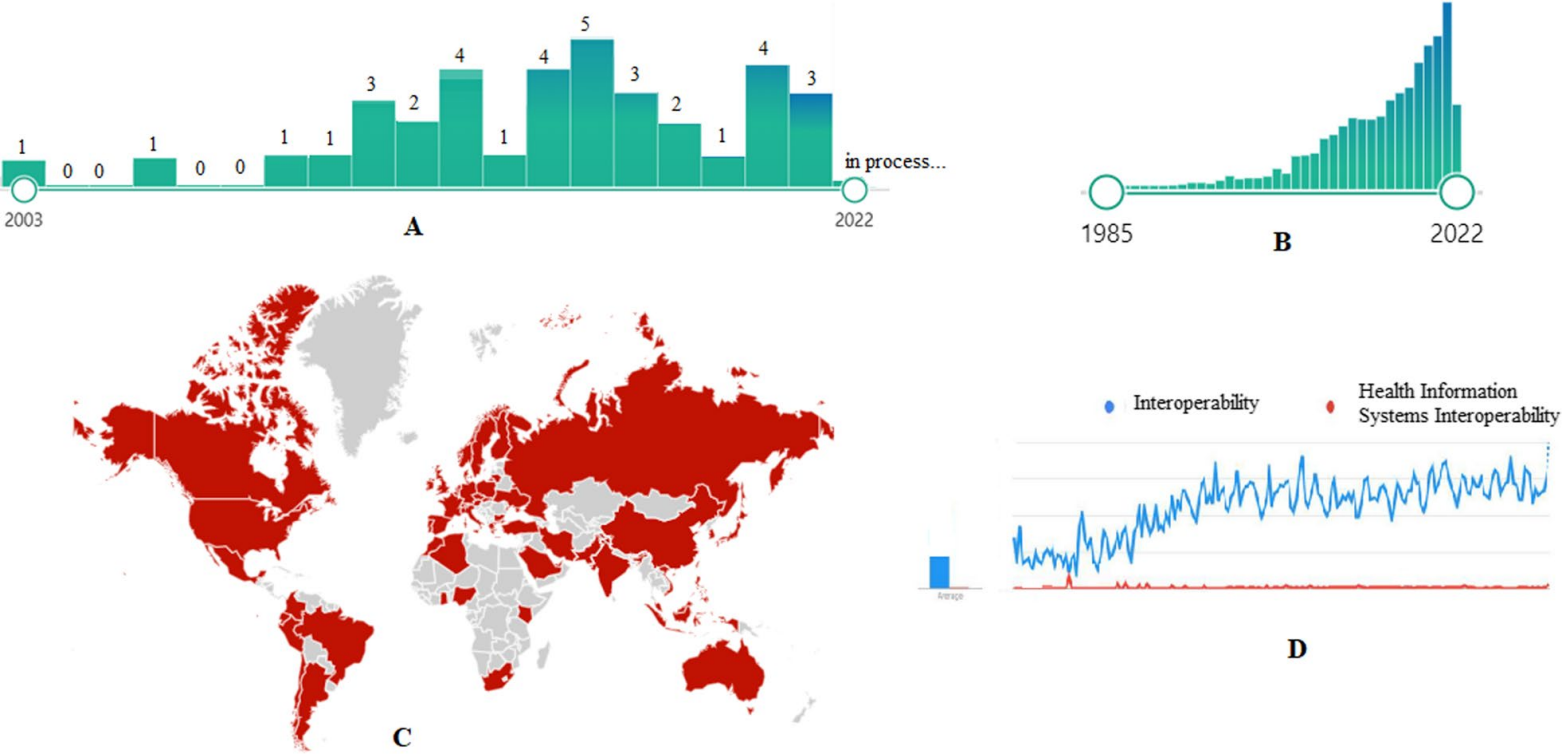
**A** The frequency of selected health information systems interoperability articles from databases based on years. **B** The growth trend of articles in the field of interoperability in databases. **C** Geographical scope in the subject of interoperability in the world based on the google search (https://trends.google.com). **D** The comparison of the google search trend of interoperability and health information systems interoperability (https://trends.google.com)

The issue of interoperability has grown exponentially in recent years and has attracted the attention of researchers around the world. Comparing the interoperability topic and health information systems interoperability shows that the extent of interoperability is much greater than the health information systems interoperability, regardless the results of the study show that this topic has been raised in the field of health information systems from 2003; and now it is one of the topics of interest to researchers (Fig. 2).

Based on the results, most of the studies were at the national level with the aim of creating interaction between clinical information systems and medical devices in order to achieve electronic health record. They belonged to the United States, Italy, Portugal, Korea and Spain, respectively. The majority of the research settings were hospitals.

The body of studies support data exchange. In addition, the processes of collecting, storing, searching, retrieving, accessing, updating, editing, and deleting were reported. Medical decision making, reimbursements, telehealth, and disease surveillance were the most commonly utilized applications, according to the mentioned process. Interoperability between systems was mostly at the semantic level and based on the Open EHR and RIM information source. More details are given in Additional file 1.

**Fig. 3:**
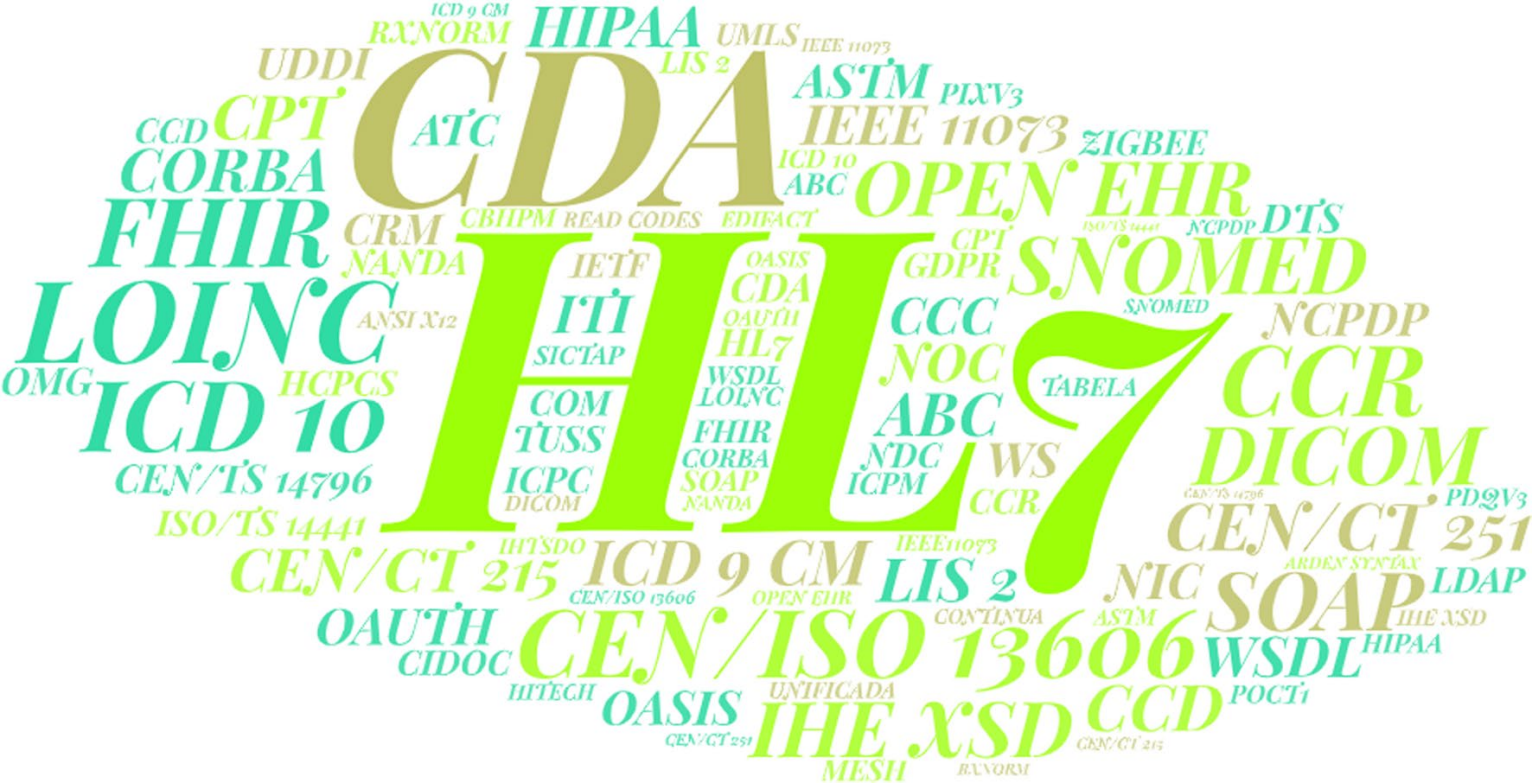
Frequent standards words cloud based on findings

**Fig. 4:**
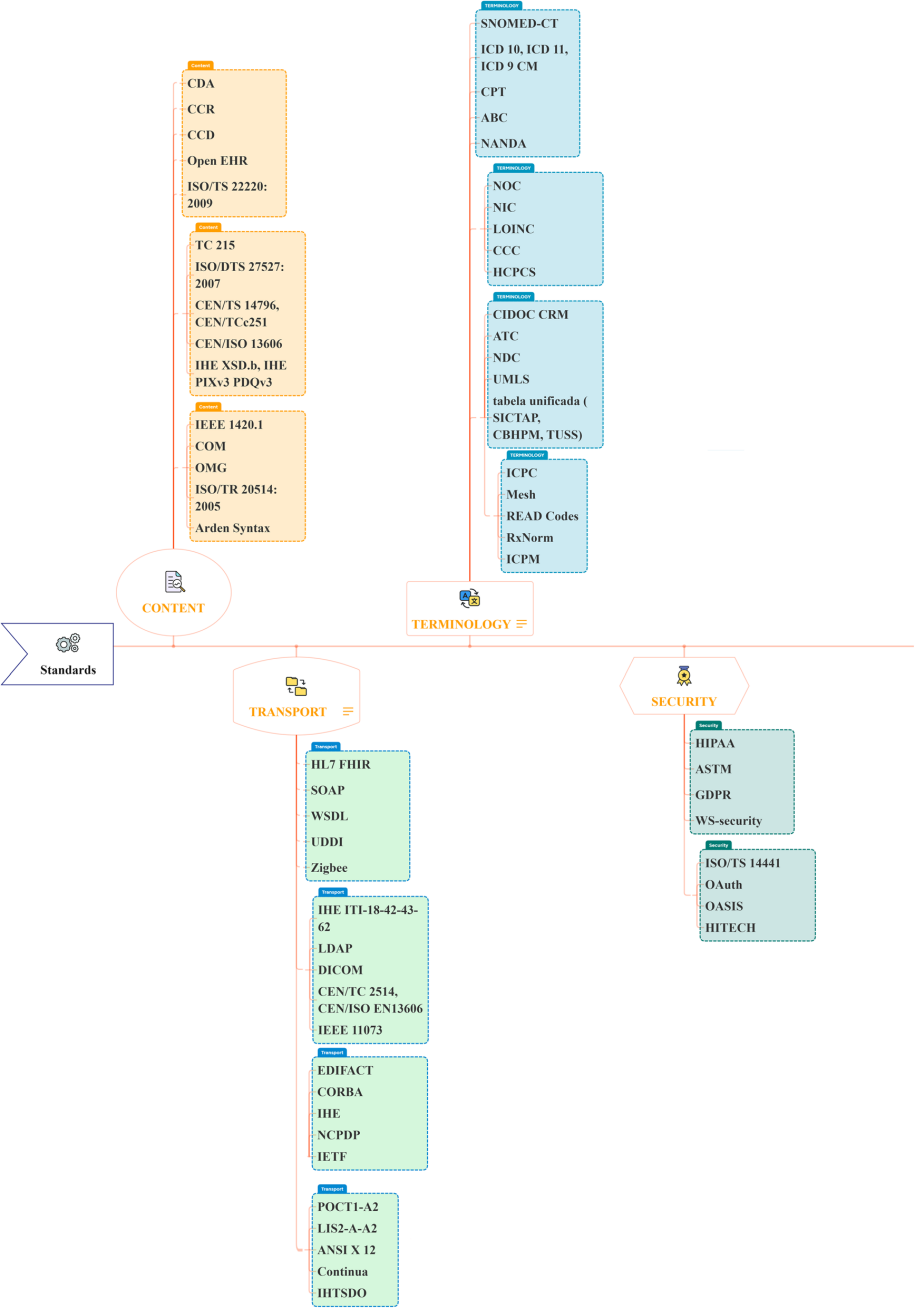
Summary of interoperability standards based on findings

**Fig. 5:**
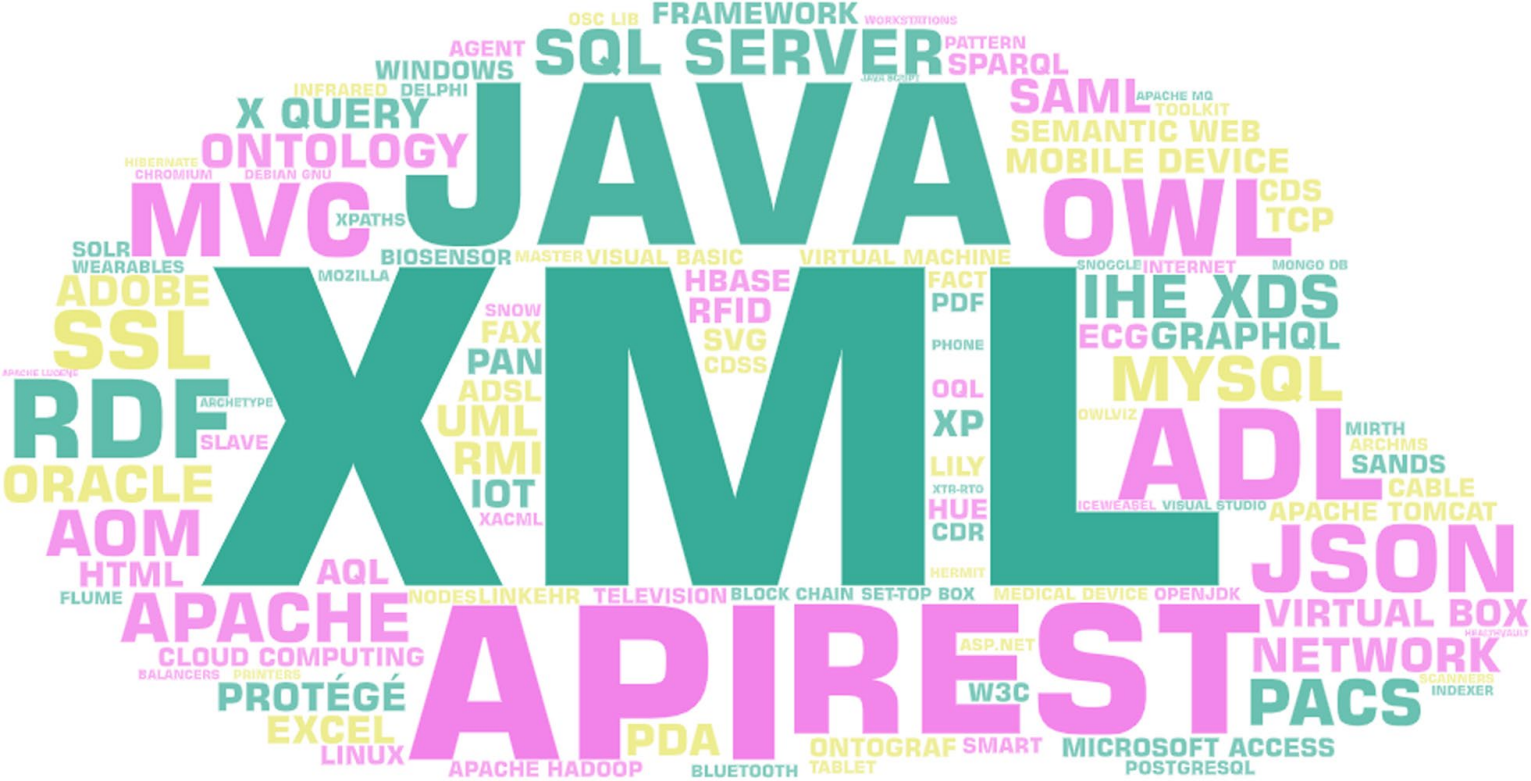
Frequent platform words cloud based on findings

The most widely used transport standards were HL7 FHIR and DICOM; the most often used content standards were CDA; the most frequently used terminology standards were SNOMED-CT, LOINC, and ICD 10; and the most commonly used security standards were HIPAA (and ASTM (Fig. 3). Figure 4 shows the requirements of interoperability implementation standards. Also XML, JAVA, SQL was the most used technologies (Fig. 5).

**Fig. 6:**
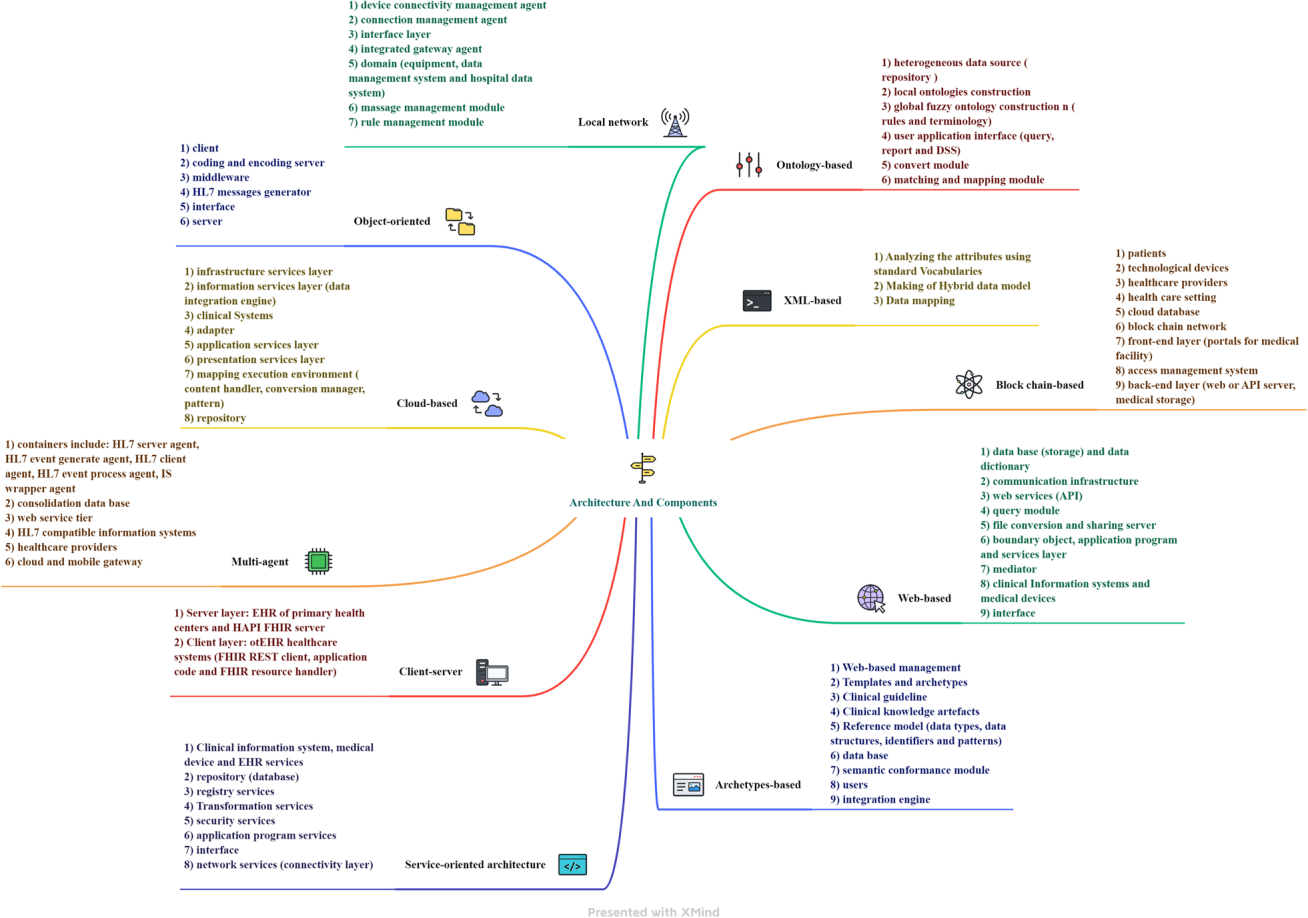
Summary of interoperability architectures components based on findings

Among the modern architectures of the world, service-oriented and web-based architecture were the most used. Figure 6 summarizes the various architectural components of the interoperability.

## Discussion

The digitalization of healthcare offers significant improvements in global health, but it is not without its own set of difficulties. Health information technology systems abound in the healthcare industry, including electronic health records, billing software, multiple portals, and individual medical equipment with their own user interfaces, to name a few [13]. Because of segregated data and legal regulations, many of these technologies can't operate together, leaving healthcare providers and personnel to undertake the manual job, which comes at a high cost. Interoperability can help in this situation. Interoperability solutions in healthcare are the key to overcoming some of the industry's most difficult challenges, and they promise to drastically cut healthcare costs [14].

Today, a lot of healthcare costs is spent on administrative. The influence of automation on operations that need time-consuming, error-prone manual work is already benefiting the industry. Interoperability solutions are already being used by health systems to assist minimize costs and medical errors by easily sharing health data among providers, payers, labs, and others [15]. Patient outcomes are improved, service delivery is streamlined, and financial performance is improved when interoperability solutions are extended across a health system. In essence, healthcare interoperability solutions provide physicians with the information they need to better coordinate care while also lowering patient healthcare costs—a win–win situation. Additionally, the data and insights can be shared with members to enhance medication adherence and chronic disease management, resulting in a higher return on investment for health plans [16].

Medical errors that result in an adverse medication event are caused by lack of information available to patients, according to a study conducted in an inpatient setting. Medical errors are the sixth biggest cause of death in hospitals, hence they are a major problem in the healthcare industry. In addition, it has been stated that medical blunders cause numerous individuals to die in hospitals each year. In addition, each year, over one million individuals are injured because of faulty health-care processes and system breakdowns [17].

Interoperability allows to save a lot of time. If a patient is unable to communicate effectively, health or care providers can fill in the gaps without the need to contact general practitioner offices or other agencies. In addition, because the medical interoperability gateway extracts and shows patient data in an existing system, providers can spend more time treating patients. When it comes to providing quality care, patient safety is paramount [18]. Clinicians' capability to access data at the point of care is critical for preventing medical errors. So, interoperability solutions that promote accuracy and accessibility lower these risks while also improving care quality. Duplication of effort and errors in patient treatment are reduced when real-time patient data is available. The information gives healthcare providers a thorough picture of a patient's medical and social history [15]. Hence, there will be no duplication of testinga between settings, and the patient will only have to tell their history once. Delays in data transport are caused by a variety of factors; however, interoperability can assist reduce these issues. Clinical decisions can be made faster and safely after viewing the medical record, reducing transfer of care, distress, and protracted stays in the hospital for patients. Coordinating care and sharing information between health and social care organizations can help determine if a patient is ready to be discharged, lowering readmissions [19].

Unlike in other industries, where digital transformation has made work easier, increasing regulatory compliance requirements, a lack of interoperability, and the sheer amount of software solutions have added to the workload for clinicians and administrators. Interoperability in healthcare reduces paperwork for employees and eliminates the need for human data entry. By supporting seamless health data sharing, interoperability solutions are smoothly taking over labor-intensive activities backlogged in systems inboxes, limiting the amount of tasks that require manual touches, reducing redundant work, and eliminating the burden placed on providers by payers [20].

Sharing healthcare data among health systems, payers, and providers improves not only the quality of care but also the efficiency with which it is given. Interoperability solutions in healthcare are easing the coordination and delivery of patient care as the sector advances toward value-based care. Approximately, healthcare interoperability allows health institutions to build the technology infrastructure needed to maximize the value of their EHR data and provide more comprehensive care [21].

The use of interoperability in the healthcare arena will allow caregivers to better understand phrases and concepts as data is transferred from one system to another while maintaining the content's meaning. Subsequently, interoperability will contribute to the development of healthcare by ensuring that communication systems receive the correct meanings of medical language. For this reason, clinicians may quickly analyze data from all collaborating systems to make diagnoses and decisions [22].

Patients trust their providers to keep clinical data private, which is why compliance is so critical for healthcare interoperability. Hospitals are balancing the need for patient health data to be available with the requirement to protect patient privacy as the number of cybersecurity assaults on healthcare institutions rises. Security procedures are used in today's healthcare interoperability solutions to ensure that data is transferred appropriately and securely certified. The fewer healthcare personnel who update patient data manually, the lower the risk of security breaches [23].

Based on results, Interoperability was more at the semantic level. In general, there are several basic levels of different levels of interoperability that have been defined in literature. These levels include:

- *Technical interoperability* At this level of interoperability, data is exchanged across systems using a communication protocol. At the plug-and-play, signal, and protocol levels, technical interoperability establishes harmonization.
- *Syntactic interoperability* Is the capacity of two or more systems to share data and services using a common interoperability protocol like the High Level Architecture [24].
- *Pragmatic interoperability* When interoperating systems are aware of one other's processes and procedures; this level of interoperability is attained. This means that the participating systems comprehend the data's use or the context in which it is used.
- *Dynamic interoperability* Two or more systems are considered to have achieved dynamic interoperability when they can understand and take advantage of state changes in the assumptions and limitations they are making over time.
- *Conceptual interoperability* When the assumptions and restrictions of a meaningful abstraction of reality are aligned, conceptual interoperability is achieved.
- *Structural interoperability* Multimedia, hypermedia, object oriented data and other forms of information is recorded.
- *Functional interoperability* Refers to the requirement for functional requirements to be delivered in a consistent, established manner.
- *Semantic interoperability* Semantic interoperability refers to the ability of two or more systems to automatically comprehend meaningful and correct information transferred in order to deliver useful results as defined by the systems' end users. Consequently, even if their instances are heterogeneously represented, that is if they are differently structured and/or use different terminology or natural language, the systems can recognize and process semantically similar information homogeneously. Semantic interoperability is distinguished from the other levels of interoperability because it assures that the receiving system understands the meaning of the sent information, even if the receiving system's algorithms are unknown to the sending system. That is why it is used more than other levels [25, 26].

HL7 FHIR were the most generally used transport standards. Given that different types of health care systems use different applications and also considering that these types of institutions need to exchange information about patients, so the interoperability of health care organizations requires interfaces between different systems use a common protocol such as HL7 [27]. FHIR is an application programming interface focused standard and next-generation interoperability standard created by HL7 that used to represent and exchange health information. FHIR has been used and recommended by many studies because it combines the features of HL7 with the latest web standards, is more secure, easy to implement, free to use, and highly flexible. FHIR is the future of data interchange in the healthcare sector, and we can say that the future is bright if we consider the numerous benefits for both the system provider and the consumer [28].

The most often used content standards were CDA. The reason CDA is so popular is that it is an XML-based standard for clinical document content that is flexible and can be read by both humans and machines. It allows the entire patient medical history to be displayed in one document, reusable in several applications, eliminates content diversity [29].

SNOMED CT is now widely used, the health industry at large recognizes that adoption of the standard must continue. The advantage of SNOMED CT in this case is that the provider can reach a common language. Clinical health records using SNOMED CT support populations by facilitating early detection of emergent health issues, community health monitoring, and quick reaction to changing clinical practices, providing precise access to pertinent data while eliminating costly duplications and errors [30].

The most well-known and widely utilized architecture nowadays is service-oriented architecture. The term "service-oriented architecture" refers to a software development methodology that allows services to communicate across platforms and languages to construct applications. A service in SOA is a self-contained piece of software that performs a specified activity. The "service concept" or "service model" of computing is implemented through service-oriented architecture. Business processes are built as software services in this architectural approach, which are accessed through a set of carefully defined application program interfaces and tied into applications via dynamic service orchestration [31].

SOA benefits organizations with features like: (1) Reusable. Services can be repurposed to create a variety of applications. Because SOA services are stored in a service repository and linked on demand, each service becomes a generalized resource available to everyone. Reusing services allows businesses to save time and money when it comes to development. (2) Simple to keep up with. Because each service is self-contained, it is simple to modify and update them without affecting other services. The running costs of an organization will also be reduced as a result of this. (3) Promotes interoperability. Platforms can effortlessly transport data between clients and services because to the adoption of a standardized communication protocol, independent of the languages they're written in. (4) High availability. On request, anyone can use the SOA facilities. (5) Increased reliability. Because small services are easier to debug than massive code, SOA produces more dependable applications. (6) Scalable. SOA enables services to run on several servers, resulting in increased scalability. Furthermore, companies can limit the amount of interaction between customers and services by employing a uniform communication protocol. Scaling apps without adding extra pressure is possible by lowering the intensity of engagement [32–34].

SOA can be implemented using any service-based technology, such as REST, WSDL and SOAP. SOA-based systems can function independently of development technologies and platforms (such as Java, XML and.NET, etc.) [35].

Without question, interoperability has a significant positive impact on healthcare. But, one of the issues in health care is the barriers and challenges that have led to a lack of interoperability between systems. In the healthcare industry, there are several standards that are often overly broad and vulnerable to local interpretation and application [36]. From there, using different standards leads to confusion. There are a number of ancient health-care systems still in use today that have limited interoperability capabilities. The issue with legacy systems is that they were built for a certain activity or facility. As an extra, many of these systems are built to prohibit compatibility with the applications of other manufacturers in order to protect market share and encourage hospital or clinic chain purchases [37]. Moreover, in contrast to most businesses, the healthcare industry still relies on stacks of handwritten notes (paper records) for patient care. This is due to the fact that most healthcare professionals are resistant to switching from a paper-based to an electronic-based system. Limited administrative and legal support for information technology and related practice changes; lack of uniformity systems from different vendors; limitations on funding of information technology and resources and privacy and security concerns are other challenges that interoperability faces [38].

Regardless of how personalized medicine is defined, it is evident that improved collaboration and data sharing are fundamental to managing the rise in complicated chronic diseases. In order to understand the underlying causes of disease and develop diagnostics and therapeutics with better efficacy and safety, access to a large quantity of diverse information from institutions must be readily available. Personalized medicine approaches can address these difficulties. By making it easier to access the data in the required formats, EHR interoperability meets the need for personalized medicine. To achieve personalized medicine, interoperable EHRs with a crucial link integrating clinical data are a necessary step. It is made easier to gather, integrate, and correlate a variety of clinical data types with patient information by offering interoperable tools and infrastructure. This connectivity can drive improvements in translational research and clinical decision support, leading to improved patient outcomes and completing the bench to bedside and back paradigm [39].

The availability of large-scale open data for drug discovery has greatly improved in recent years due to the expansion of data repositories, particularly those with chemical and pharmacological data sets. A typical research project in computational drug discovery uses a variety of software, programs, and tools to read input files, pre-process data, perform one or more computations, and do post-analysis. Pre-processing and connecting the outputs of one software or tool as input to another software or tool would probably be required for this. Such an undertaking can be challenging and necessitate manual pre-processing of the output and input files. If system or tool developers also take into consideration the real-world use case situation relative to the interoperability of input/output files for various software programs and tools, this problem might be resolved [40].

Manufacturing architectures have evolved into integrated networks of automation devices, services, and businesses thanks to recent developments in manufacturing technologies including cyber-physical systems, the industrial internet, artificial intelligence, and machine learning. The rising requirement for interoperability at all levels of the manufacturing ecosystem is one of the difficulties that have come about as a result of this growth. The range includes everything from shop floor software, devices, and control systems to web-based cloud platforms that offer a variety of services on demand. Thus, a successful interoperability implementation in smart manufacturing will lead to efficient communication and error-prone data interchange between devices, users, systems, and platforms. The architecture and platforms that are utilized by machines and software programs present a considerable obstacle to this. Industry-specific interoperability and their corresponding logical semantics can help us comprehend the topic better [41].

A study by de Mello et al. has been conducted in 2022 [42], in which the interoperability requirements of health records have been addressed only at the semantic level and with an approach from the perspective of standards, and other levels of interoperability as well as other requirements such as scope of use, architectures, components, platforms, data sources, involved systems and processes, as well as advantages or challenges, have not been included, while this study has all of them and the audience has the possibility to understand the reason for their application in addition to being familiar with these requirements. Another advantage of the current study is the wide time range of the search, while the mentioned study only supports the 10-year time range from 2010 to 2020. Also, another added value of our study is the management discussions and its prospective perspective on interoperability. Finally, these two researches are done with two different views and provide the audience with a complete view together.

## Conclusions

Interoperability in the field of health care has not yet been reached. The lack of interoperability between healthcare systems reinforces the information silos that exist in today's paper-based medical files, resulting in private ownership over health data. Consequently, healthcare costs have risen, patient care quality has deteriorated, and the ability to integrate patient data across healthcare systems has been compromised. Therefore, it can be said, the findings of this study could serve as strategic starting points for further integration of interoperability principles into the healthcare sector and more efficient ICT integration, particularly in countries where e-health planning and development is still in its early stages. Added to that, the issues mentioned could encourage much-needed change in the information systems development field and promote further steps toward general interoperability in the national and international healthcare environments. Nevertheless, promoting interoperability between information systems that deal with sensitive information takes a significant amount of time and requires a large multidisciplinary expert team.

### Limitations

Due to the fact that interoperability is a very broad subject and includes various aspects and the scope of recent studies in this field is very large, therefore, in the present study, mostly technical, informatics and management issues of interoperability have been discussed. However, legal and organizational issues are among the most important bottlenecks in interoperability, which were not covered in this study. Therefore, it is suggested that each of the legal and organizational areas be addressed separately in future studies. Also, lack of access to some articles due to the lack of access to their full text and their non-English language was another limitations of the study.

## Supplementary Information

**Additional file 1.** Details of the selected studies in this review.

## Data Availability

The dataset supporting the conclusions of this article is included within the article (and its additional file).

## Abbreviations

ICT: Information and communications technology
EHR: Electronic health record
EMR: Electronic medical record
PHR: Patient health record
SNOMED-CT: Systematized nomenclature of medicine-clinical terms
ICD: International classification of diseases
CPT: Current procedural terminology
ABC: Alternative billing codes
NANDA: North american nursing diagnosis association
NOC: Nursing outcomes classification
NIC: Nursing interventions classification
LOINC: Logical observation identifiers names and codes
HCPCS: Healthcare common procedure coding system
CCC: Clinical care classification
CDA: Clinical document architecture
HL7: Health level seven
HIPAA: Health insurance portability and accountability act
PC: Personal computer
XML: Extensible markup language
SOA: Service-oriented architecture
SOAP: Simple object access protocol
UDDI: Universal description discovery and integration
WSDL: Web services definition language
PDA: Personal digital assistant
ADSL: Asymmetric digital subscriber line
RMI: Remote method invocation
NHIN: Nationwide health information network
SANDS: Service-oriented architecture for NHIN decision support
REST: Representational state transfer
RFID: Radio frequency identification devices
PAN: Personal area network
CRM: Conceptual reference model
ATC: Anatomical therapeutic chemical
AIC: Akaike information criterion
FHIR: Fast healthcare interoperability resources
SOAP: Simple object access protocol
IHE: Integrating the healthcare enterprise
ITI: Information technology infrastructure
IHE XDS: Integrating the healthcare enterprise extended data services
RM: Reference model
ADL: Archetype definition language
SAML: Security assertion markup language
AOM: Archetype object model
ISO: International organization for standardization
LDAP: Lightweight directory access protocol
DICOM: Digital imaging and communications in medicine
NDC: National drug code
GDPR: General data protection regulation
TCP: Transmission control protocol
API: Application programming interface
IOT: Internet of things
HIS: Hospital (Health) information systems
WSDM: Web service of data mediation
UMLS: Unified medical language system
EDIFACT: Electronic data interchange for administration, commerce and transport
RIM: Reference information model
CDR: Clinical documents repository
ICPC: International classification of primary care
AQL: ArangoDB query language
SQL: Structured query language
JSON: JavaScript object notation
CIS: Clinical information system
CCR: Continuity of care record
PDF: Portable document format
SVG: Scalable vector graphics
URL: Uniform resource locator
CCD: Continuity of care document
LIS: Laboratory information systems
CORBA: The common object request broker architecture
OMG: Object management group’s
COM: Component object model
OWL: Web ontology language
RDF: Resource description framework
DSS: Decision support system
OQL: Object query language
HTML: Hyper text markup language
NCPDP: National council on prescription drug programs
IETF: Internet engineering task force
ASTM: American society for testing and materials
Open MRS: Open medical record system
HTTP: Hypertext transfer protocol
OAuth: Open standard for authorization
PACS: Picture archiving and communication systems
CDS: Clinical decision support
Continua: Continua health alliance
UML: Unified modeling language
ANSI: American national standards institute
HITECH: Health information technology for economic and clinical health
CORBA: Common object request broker architecture
OASIS: Organization for the advancement of structured information standards
SSL: Secure socket layer
HTTPS: Hypertext transfer protocol over secure socket layer
XACML: Extensible access control markup language
SAML: Security assertion markup language
