# Supplementary material for: Interoperability of heterogeneous health information systems: a systematic literature review
Source: BMC Med Inform Decis Mak. 2023 Jan 24;23:18. doi: 10.1186/s12911-023-02115-5 (PMC9875417; doi:10.1186/s12911-023-02115-5)
Supplement: Supplementary file 1 — Additional file 1. Details of the selected studies in this review. [file 12911_2023_2115_MOESM1_ESM.docx]

Appendix 1. Details of the studies selected in this review

| **Authors and Year** | **Country** | **Aim of research** | **Interoperable systems** | **Architecture and components** | **Setting** | **Included processes** | **Used standards** | **Platform and techniques** | **Extent of implementation** | **Level of interoperability** | **Information resource** | **Key findings** |
| --- | --- | --- | --- | --- | --- | --- | --- | --- | --- | --- | --- | --- |
| Kim HS, et al. 2011 | United States | Design and development of an electronic claim system based on an integrated EHR platform by integrating various medical standard technologies. | EMR, PHR and insurance system | Web service  1) storage layer  2) boundary object layer  3) application program layer  4) user interface layer | Ambulatory care centers | Data entry, searching, removing, editing, exchange, mapping and linkage between terminologies, clinical decision-making support and calculation of reimbursement charge | - Terminology: SNOMED-CT, ICD, CPT, ABC, NANDA, NOC, NIC, LOINC, HCPCS and CCC  - Content: CDA  - Transport: HL7  - Security: HIPAA | - Hardware: server and PC  - Technology: XML and Java | National | Functional | Centers for Medicare and Medicaid services | 1. Prevents physician malpractice by suggesting proper treatments according to patient diagnoses.  2. Provides documents for reimbursement and submits claim documents to insurance organizations electronically, easily and quickly. |
| Plácido GR, et al. 2011 | Portugal | Proposes an architecture that aims to answer the needs of interoperability between heterogeneous systems. | Health information systems, mobile devices, biosensors and a set of primitives such as medical devises | Service-oriented architecture  1) procedural layer  2) documental layer | Hospitals, primary care centers and medical emergency centers | Documentation, exchange of information, medication and telemedicine | - Transport: SOAP, UDDI, WSDL, Zigbee | - Hardware: server, mobile device, biosensor, set-top box, PDA, medical devices, ADSL, cable and television  - Software: Java RMI  - Technology: SANDS, REST, Bluetooth, RFID, internet network, PAN and infrared | National | Structural | Portugal national health service | Enables the ubiquitous access using mobile devices and web services with the objective of providing a better medical assistance in and outside health institutions. |
| Ciampi M, et al.2016 | Italy | Presents the architecture for the interoperability of regional EHR systems. | Regional EHR systems | Service-oriented architecture  1) EHR services  2) repository  3) registry  4) cross-border services  5) security  6) public connectivity system | All health care settings | Exchange of health information, searching, retrieving, creating or updating and invalidating | - Content: CDA  - Terminology: CIDOC CRM, ICD9-CM, LOINC and ATC  - Transport: HL7 FHIR, IHE ITI-18-42-43-62 and SOAP | - Technology: IHE XDS and XML | National | Functional | National research council of Italy and the agency for digital Italy | Interoperable EHR are able to improvement of the quality of care and reduction of costs. |
| Chen X, et al. 2015 | Ireland | Introduces a generalized identity reference model to support the exchange of instances of demographic identities. | EHR systems | Archetypes-based  1)reference model  2) archetypes | All health care settings | Exchange of demographic identities | - Transport: LDAP and HL7  - Content: open EHR, ISO/TS 22220:2009 - TC215, ISO/DTS 27527:2007 - TC215, CDA, CEN/TS 14796 | - Technology: AOM, ADL and SAML | National | Structural | HL7 RIM and ISO demographics | Identity management systems enable safe and convenient identity exchange between different domains and according to different standards. |
| Centobelli P, et al. 2021 | Italy | The goal of study is to create an integrated view that will collect data from patients' medical records, based on the use of sensors from different wearable devices and otEHR sources. | Health care systems and sensors | Block chain-based  1) patients  2) technological devices  3) healthcare providers  4) health care setting  5) cloud database  6) block chain network | Laboratories, radiology, pharmacies and hospitals | Record, update and share of health data | - Transport: DICOM and HL7 FHIR  - Security: GDPR and HIPAA  - Terminology: ICD 10, ICD 11, NDC, SNOMED-CT and LOINC | - Hardware: wearables and mobile  - Software: GraphQL  - Technology: block chain, REST, TCP, API, IOT and cloud computing | National | Structural | N/A | Advantage of this approach for healthcare professionals is that their patients' health information, is always up to date, beyond the ability to extract medical statistics to increase treatment quality. |
| Mantas J. 2020 | Burkina Faso | This paper presents a semantic interoperability architecture, which is based on a mediation approach. | Between many HIS | Web services  1) Web service of data mediation  2) User interface  3) Knowledge base  4) Referential ontology  5) Mediator | Hospitals | Exchange semantically data | - Transport: DICOM, HL7, CEN TC2514, IEEE 11073 and EDIFACT  - Content: CEN/ISO 13606 and CDA  - Terminology: SNOMED-CT, UMLS and LOINC | - Software: semantic web tools  - Technology: ontology and XML | National | Semantic | RIM | One of the main problems encountered in the implementation is the identification of referential ontologies. |
| Oliveira EC. 2017 | Brazil | This paper presented a list of specifications of the solutions implemented in the establishment of the Brazilian national EHR. | PHR and national EHR | Service-oriented architecture  1) Demographic services  2) Health Service Bus  3) Security services  4) Semantic framework  5) Clinical information repository services | Primary care centers and hospitals | Exchange, store, index, retrieve, search and access data | - Content: open EHR, IHE XSD.b and IHE PIXv3 PDQv3  - Transport: SOAP, HL7 and WSDL  - Terminology: SNOMED-ICD10, tabela unificada (SICTAP, CBHPM, TUSS), ICPC and LOINC  - Security: WS-security | - Software: Oracle  - Technology: REST, CDR, AQL and XML | National | Semantic | Open EHR which were customized with the Brazilian EHR | Interoperability requirements allow the implementation of web services that are interoperable with EHR internal and external health systems. |
| Janaswamy S and Kent RD. 2016 | Canada | This study propose an original algorithm directed at mapping unstructured data and performing semantic integration to form a uniform interoperable system. | EMR or EHR systems | XML-based  1) Analyzing the attributes using standard Vocabularies  2) Making of Hybrid data model  3) Data mapping | Hospitals | Data storage, access, exchange and extraction | - Transport: HL7  - Terminology: LOINC | - Software: Java software platform, Microsoft access database, MySQL,  - Technology: API and XML | National | Semantic | N/A | This approach caused to lay the foundation for providing a cost effective, secure and reliable system for real time clinical data acquisition required for analysis. |
| Hidayat IF and EHRmanto BR. 2020 | Indonesia | The objective of this study is to implement the EHR interoperability in primary health centers. | Primary health centers EHR systems | Client server  1) Server layer: EHR of primary health centers and HAPI FHIR server  2) Client layer: otEHR healthcare systems (FHIR REST client, application code and FHIR resource handler) | Primary health centers | Create, read, update, delete and exchange of data | - Transport: HL7 FHIR  - Terminology: ICD 10, ICD 9 CM, SNOMED-CT and CPT  - Content: CDA | - Technology: API, json and java and REST | Local | Semantic and syntactic | FHIR resource | The result showed that HL7 FHIR implementation supports the syntactic and sematic interoperability in primary health centers that can provide quality data to support the referral system, analysis purposes and health decision making. |
| Miranda M, et al. 2012 | Portugal | This paper aims to propose a multi-agent based architecture for the implementation of interoperability in healthcare environment. | Between HIS | Multi-agent  1) containers include: HL7 server agent, HL7 event generate agent, HL7 client agent, HL7 event process agent, IS wrapper agent  2) consolidation data base  3) web service tier  4) HL7 compatible information systems | Hospitals | Exchange of data | - Transport: HL7  - Content: open EHR | - Software: Java agent development framework  - Technology: XML | National | Syntactic | N/A | The most important characteristic of this architecture is that instead of a mesh of end-to-end system communication or a major centralization of processing, this paradigm is by nature distributed but allows a consolidation of processual and clinical validation of information. |
| Garde S, et al. 2009 | Sweden | This paper aims to improve semantic interoperability of EHRs. | Between EHRs | Archetype-based  1) Web-based management  2) Templates and archetypes  3) Clinical guideline  4) Clinical knowledge artefacts  5) Reference model | All health care settings | Information sharing and decision support | - Content: open EHR, ISO 13606, CCR | N/A | National | Semantic | Open EHR | Interoperability play a major role in provision of quality care and safety information sharing. |
| Marcheschi P, et al. 2006 | Italy | The purpose of this work is to reuse existing ECG devices into the EHR data distribution, and at the same time to be totally independent from the development platform, from the programming language and the operating system used. | Between ECG system and EHRs | Service-oriented architecture  1) common services (registry)  2) client infrastructure (Document Source)  3) network services infrastructure | All health care settings | Exchanging document | - Transport: HL7, DICOM and IEEE  - Content: CDA  - Terminology: LOINC | - Software: Adobe PDF, Adobe SVG and PACS  - Hardware: ECG , XML and Holter monitoring device, server and computer | National | Functional | HL7 RIM | This approach can be useful to integrate legacy ECG acquisition systems in an effective simple and low cost way, opening the possibility to create a central node for distribution and archiving biomedical signals. |
| Andersen B, et al.2015 | Germany | The purpose of this work is to developed a software component that increase semantic interoperability between medical devices and CIS. | Surgical devices and CIS | service-oriented architectures  1) device observation reporter  2) medical device network communication  3) clinical IT network communication  4) CIS  5) medical device  6)Transformation | Hospitals | Data collection, transformation and reporting | - Transport: HL7, and ISO/IEEE11073  - Content: CDA | - Software: Debian GNU/Linux 7.8 operation system, Oracle VM Virtual Box 4.3.18, OpenJDK 7u75, PostgreSQL 9.1, Apache Tomcat 8.0.14, Iceweasel 31.6, Chromium 41.0 and OSC Lib 0.90b  - Hardware: server  - Technology: Model-view-controller pattern, Java and virtual machine | National | Semantic | German federal ministry of education and research | This solutions could incorporate clinical knowledge to autonomously select signal combinations and generate reports of diagnostic and interventional procedures, thus saving time and effort for surgical documentation. |
| Franček P, et al. 2015 | Croatia | This paper aims to integrating PHR and EHR using HL7 CCD. | EHR and PHR | Cloud-based  1) EHR  2) PHR  3) local clinical systems  4) adapter | All healthcare settings | Data sharing | - Content: CDA and CCD  - Transport: DICOM | - Software: PACS  - Hardware: server, computer, mobile  - Technology: API , XML, Java and REST | National | Semantic | HL7 RMIM | Using CCD has been proven a smart choice because development and implementation of interoperability adapter was easy and straight forward process. Preserving and sharing medical images in EHR is accomplished by storing a URL link in the result part of observation. |
| Kopanitsa G and Ivanov A. 2018 | Russia | The goal of the paper is to present an implementation of FHIR profiles and data exchange infrastructure to automate HIS – LIS workflows. | Laboratory systems and HIS | Web-based  1) LIS  2) HIS  3) interface | Laboratories and hospitals | Data exchange | - Terminology: LOINC  - Transport: HL7 FHIR and CEN/ISO EN13606  - Content: CCR and open EHR | - Technology: API and REST | Local | Semantic | open EHR | The presented approach showed a high efficiency in a data exchange process. |
| Pintea R,et al. 2010 | Romania | The paper presents an interoperable software solution developed to support the activities in the cardiology department of a big hospital. | Cardiology department systems and HIS | service-oriented architectures  1) hospital network  2) database  3) interface  4) HIS  5) cardiology department systems | Hospital | Storing and exchanging data and metadata | - Transport: HL7, SOAP and DICOM  - Content: IEEE 1420.1 | - Software: XML, SQL server and Java  - Hardware: server, computer, smart phone and tablet  - Technology: XML and Java | Local | Semantic | Basic interoperability data model | This approach will allow an easier and faster configuration of the system, by using the interoperability and medical standards. Using this system will improve the work process and the knowledge of the nurses but EHR is the problem of limited resources. |
| Amr MF. 2020 | Morocco | This article is presenting a hybrid extensible model that allows HIS to interoperate with each otEHR through the modeling of the "request for medical care" web service. | Between HIS | Web service  1) data base in cloud  2) medical sectors  3) web service  4) query module  5) file conversion server  6) email server  7) file sharing system | Hospitals | Data collection, consolidation, exchange and storage | N/A | - Hardware: fax and server  - Technology: cloud computing, XML and JSON | National | Functional | N/A | A web service has been used to facilitate communication between the HIS and the data base hosted in the cloud. |
| Vargas B and Ray P. 2003 | Australia | This paper develops of a solution for the interoperability of CIS for a hospital. | Between CIS | object-oriented  1) client  2) coding and encoding server  3) middleware  4) HL7 messages generator  5) interface  6) server | Hospital | Exchange of data | - Transport: HL7, DICOM and CORBA  - Content: OMG and COM | - Software: java toolkit, Delphi, java virtual machines and SQL  - Technology: API, visual basic and C++ | Local | Semantic | N/A | It is possible to implement the interoperability of HIS applications through CORBA, Java and HL7 that can lead to a numerous advantages. |
| Adel E, et al. 2019 | Egypt | This paper proposes a fuzzy-ontology framework that could integrate most existing EHR different data models. | Between CIS | Fuzzy ontology architecture  1) heterogeneous data source  2) local ontologies construction  3) global fuzzy ontology construction n (rules and terminology)  4) user application interface (query, report and DSS) | All healthcare settings | Information sharing | - Transport: HL7  - Content: CEN/ISO 13606 and open EHR  - Terminology: SNOMED-CT | - Software: protégé, Excel, MySQL, OntoGraf, ArchMS, XTR-RTO, xml2owl and X2OWL  - Technology: OWL, RDF, ADL and XML | National | Semantic | Open EHR | This framework is a step towards improving healthcare performance and reducing both human mediation and data losses. |
| Bhalla S, et al. 2017 | United States | The goal is to present an approaches in understanding some current and challenging concepts in e-health informatics. | Between EMR | Archetypes-base  1) reference model (data types, data structures, identifiers and patterns)  2) conceptual model (archetypes and templates) | All healthcare enterprises | Exchange of data | - Transport: HL7  - Content: CEN/ISO 13606 and open EHR  - Security: ISO/TS 14441 | - Software: Mongo DB, XQuery, SQL, OQL, W3C and XPaths  - Technology: ADL, XML and JSON | National | Semantic | Open EHR | Successful handling of interoperability challenges will lead to improved quality in healthcare by reducing medical errors, decreasing costs, and enhancing patient care. |
| Angula N and Dlodlo N. 2018 | Namibia | The aim of this study is to find a framework that can enable the semantic interoperability of data in heterogeneous HIS. | Between HIS | Web-based  1) HIS  2) middleware  3) registry  4) repository  5) dashboard | Hospitals | Exchange important disease-surveillance information | - Transport: HL7, IHE, DICOM, NCPDP and IETF  - Terminology: LOINC  - Content: ISO and CDA  - Security: ASTM | - Software: SQL  - Technology: XML and HTML | National | Semantic | HL7 massaging structure | This approach cause that they can share and exchange disease surveillance data for informed decision-making. |
| Kasthurirathne SN, et al. 2015 | United States | This study proposes an integration path that uses the FHIR API, which is integrated into the main Open MRS platform. | Between EMR | Web services  1) FHIR web layer (controller and resource)  2) FHIR API layer  3) service layer | All healthcare enterprises | Data exchange | - Transport: HL7 FHIR  - Content: CDA  - Security: OAuth  - Terminology: LOINC | - Software: Mozilla  - Technology: REST, XML, JSON, HTTP and API | National | Semantic | Open MRS | These efforts demonstrate the adoption of an emerging FHIR standard that is seen as a replacement for both HL7 Version 2 and 3. |
| Park KS, et al. 2013 | Korea | The purpose of study was to design an integrated data management system to ensure data interoperability between mobile equipment and the existing hospital data system. | Mobile equipment and the existing hospital data system | Local network  1) device connectivity management agent  2) connection management agent  3) interface layer  4) integrated gateway agent  5) domain (equipment, data management system and hospital data system)  6) massage management module  7) rule management module | Hospital | Data exchange | - Transport: HL7, POCT1-A2 and LIS2-A,  LIS2-A2 | - Software: Visual Studio, MS SQL, .Net Framework 2.5, Microsoft Windows XP and Microsoft Windows Server  - Hardware: server  - Technology: C# language | Local | Structural | N/A | In comparison with the existing system, the data management system facilitated integration by improving the result receiving time, improving the collection rate, and by enabling the integration of disparate types of data into a single system. |
| Jabbar R, et al. 2020 | Qatar | This work proposes a block chain framework for enhancing data interoperability and integrity regarding EHR-sharing. | Between HIS | Block chain-based  1) HIS- front-end layer (portals for medical facility)  2) HIS-back-end layer (web or API server, medical storage)  3) block chain layer (EtEHReum private cloud)  4) access management system | Hospitals | Collecting, storing and sharing data | - Terminology: ICD 10  - Transport: DICOM | - Software: SQL server, CDS and PACS  -Hardware: network hardware, printers, workstations, servers and scanners  -Technology: API | Local | Semantic | N/A | This framework fully ensures support for cost-effectiveness, scalability across large populations of patients, structural interoperability at the minimum, user identification and authentication, and Turing-complete operations. |
| Berges I, et all. 2011 | Spain | In this paper, we present a proposal that smooth out the way toward the achievement of semantic interoperability of medical diagnosis statements of EHR. | Between HIS | Ontology-based  1) repository of each healthcare institution  2) application ontologies (convert DB to ontology module )  3) canonical ontology (mapping ontology module) | All healthcare enterprises | Data sharing | - Content: open EHR, ISO 13606 and CDA  - Transport: HL7  - Terminology: SNOMED-CT and LOINC | - Technology: OWL, XML and ADL | National | Semantic | Open EHR | Their descriptions are independent of languages and technology aspects used in different organizations to represent EHRs. |
| Martínez-Villaseñor M, et all. 2016 | Mexico | This paper proposed to leverage the interoperability between standards through the mediation of a ubiquitous user model and an automatic process of concept alignment. | Between PHR | Ontology-based  1) profile suppliers (source document)  2) matching module  3) profile consumer (input requirement) | All healthcare enterprises | Data sharing | - Content: ISO/TR 20514, ISO 13606 and CDA  - Transport: HL7 , CEN/TC 251 and DICOM | - Software: Microsoft HealthVault  - Technology: API, XML, JSON and RDF | National | Semantic | Open EHR | Results prove that the process is making sense in the schema matching decisions, in many cases semantic interoperability is hard and the human effort to mend ambiguities is too much. |
| Beštek M and Stanimirović D. 2017 | Slovenia | The main aims of the paper comprise the characterization and examination of the potential approaches regarding interoperability. | Between EMR | Multi-approach  1) national E-health  2) healthcare provider  3) cloud gateway  4) mobile gateway  5) patient device | All healthcare enterprises | Health information exchange | - Content: open EHR  - Transport: Continua, HL7 FHIR and IHE  - Terminology: SNOMED-CT, ICD 10 and LOINC | - Technology: REST, XML, JSON and ADL | National | All level | Open EHR | Many changes are needed in the field of IS development and EHR steps towards interoperability in the national and international healthcare environment. |
| Sachdeva S and Bhalla S. 2012 | Japan | This study aims to provide different approaches in understanding some current and challenging concepts in interoperability. | Between EMR | Archetype-based  1) database schema  2) clinical model  3) user  4) expert  5) semantic conformance (ADl, archetype and template language)  6) reference model | All healthcare enterprises | Data exchange | - Content: open EHR, CDA, CEN/TC251 and ISO 13606-2  - Transport: HL7, DICOM, IEEE, IHTSDO and IHE  - Terminology: SNOMED-CT, ICPM, ICD 10 and LOINC  - Security: ASTM | - Technology: XML, HTML, UML, JSON and ADL  - Software: SQL server and XQuery | National | Semantic | Open EHR | Successful handling of challenges will lead to improved quality in healthcare by reducing medical errors, decreasing costs, and enhancing patient care. |
| Marcos M, et all. 2013 | United States | This study deal with the interoperability problem of CDSSs and EHRs by exploiting the dual-model methodology | Between CDSS and EHR | Archetype-based  1) reference model (generic properties and structure of information)  2) archetypes repository (clinical concepts)  3) link EHR integration engine  4) clinical database  5) link EHR editor engine (mapping, archetypes and terminology)  6) link EHR transformation engine | Hospitals | Data exchange and decision support | - Transport: HL7 and CEN/ISO EN13606  - Content: CDA, open EHR and CCR  - Terminology: SNOMED-CT | - Technology: AOM, OWL, ADL and XML  - Software: SQL server and XQuery | Local | Semantic | Reference model | The utilization of archetypes not only has proved satisfactory to achieve interoperability between CDSSs and EHRs but also offers various advantages, in particular from a data model perspective. |
| Bahga A and Madisetti VK. 2013 | United States | This paper describe the high level design of Cloud Health Information Systems Technology Architecture and the approaches for semantic interoperability, data integration and security. | Between EMR | cloud-based  1) infrastructure services layer  2) information services layer (data integration engine)  3) application services layer (such as terminology services etc.)  4) presentation services layer (healthcare applications) | All healthcare enterprises | Store and transfer and access data | - Transport: HL7, DICOM, ANSI X12 and NCPDP  - Content: CDA, CCR and CCD  - Security: HIPAA, HITECH and OAuth | - Software: MySQL, Mirth Connect, Java software platform, load balancers, Hadoop master and oracle  - Technology: API, cloud computing, XML and REST  - Hardware: server, PC, slave nodes and network equipment | National | Semantic | Open EHR reference model and archetype model | This approach supports advanced security features and addresses the key requirements of HIPAA and HITECH and has better interoperability, scalability, maintainability, portability, accessibility and reduced costs as compared to traditional client-server EHR systems. |
| Ciampi M, et al. 2013 | Italy | This paper presents an architecture for federated HIS in a secure manner, and its concrete implementation. | Between HIS | Service oriented architecture  1) connectivity layer (connection infrastructure)  2) component layer (infrastructural components)  3) business layer (application services) | All healthcare enterprises | Exchange of medical data | - Transport: HL7, DICOM and CORBA  - Content: open EHR, CDA and CEN/ISO EN 13606  - Security: OASIS | - Technology: XML, SSL, Java, HTTPS and IHE XDS  - Software: apache MQ | National | Semantic | RIM | The results showed that the proposed method was of high quality when sending, retrieving and informing documents. |
| del Carmen Legaz-García M, et al. 2016 | Spain | This paper describe an approach for the interoperability and that enables the secondary use of clinical data. | Between clinical systems and EHR | Web based  1) acquisition layer (convert EHR data to archetype-ontology)  2) repositories layer (primary data about the clinical archetypes and extracts)  3) exploitation layer (services for the exploitation of the archetypes and the EHR data) | Hospitals | Data exchange | - Content: CEN/ISO 13606 and open HER  - Transport: HL7 FHIR  - Terminology: SNOMED-CT | - Technology: semantic web, ontology, ADL, OWL, XML, RDF and apache Lucene API  - Hardware: computer  - Software: archetype management system, SPARQL, semantic web integration tool, ontology pre-processing language version 2, LinkEHR and Protégé | Local | Semantic | open EHR | Results showed the potential of semantic web technologies for the management and exploitation of archetypes and EHR data. |
| Khan WA, et al. 2014 | Korea | study propose an adaptive adapter interoperability engine mediation system, that arbitrates between HISs for accurate and seamless information exchange. | Between HIS | Cloud based  1) consumer applications  2) mapping execution environment (content handler, conversion manager, pattern)  3) mapping authoring environment (mediation bridge ontology, accuracy mapping engine, repository) | Hospitals | Information exchange | - Content: CDA, Arden Syntax, CEN/ISO 13606 and open HER  - Transport: HL7 | - Technology: ontology, CDSS and UMLS  - Software: LinkEHR | Local | Semantic | RIM | 1. The transformation process achieved over 90 % of accuracy level in conversion process.  2. The proposed mediation system improves the overall communication process between HISs and healthcare services. |
| Mukhiya SK and Lamo Y. 2021 | Norway | The aim of the study was to develop a system to communicate between heterogeneous healthcare systems based on HL7 FHIR. | Between EHR | Service oriented architecture  1) open MRS server  2) resource server  3)authorization server  4) patient app  5) provider app  6) translator | Hospitals | Information exchange | - Transport: HL7 FHIR  - Content: CDA and Open HER  - Terminology: SNOMED-CT, RxNorm, ICD 10 and LOINC  - Security: OAuth | - Software: GraphQL, JavaScript, MySQL, Apache Tomcat, Hibernate and SQL server  - Technology: REST, API, XML, JSON and HTTP  - Hardware: servers | Local | Technical and semantic | Open MRS | The presented approach establishes secure communication between the EHRs and provides accurate mappings that enable timely health information exchange between EHRs. |
| Cassavia N, et all. 2016 | Italy | This paper proposes an architecture for supporting interoperability in healthcare systems by exploiting Big Data techniques. | Between EMR | Web service  1) repository  2) knowledge discovery module  3) service modules (collect, aggregate and forward data- data cleaning and data mapping and so on) | Hospitals | Medical data exchange | - Transport: HL7, CORBA and DICOM  - Content: CDA and CEN/ISO 13606  - Terminology: SNOMED-CT, RxNorm, ICD 10 and LOINC  - Security: OASIS | - Technology: XML, IHE XDS, SSL, SAML and XACML  - Software: Apache Hadoop, Flume, HBase, Solr, Lily HBase Indexer and Hue | National | Syntactic and Semantic | Open EHR | The proposed approach was able to improve EHR data access efficiency and reduce costs. |
| Ebietomere EP, et al. 2021 | Nigeria | This paper explicates how an interoperable EMR ontology was crafted for a tertiary health facility. | Between EMR | Ontology based  1) extraction of concepts  2) cleaning of data  3) ontology creating  4) evaluation of ontology  5) data mapping | Hospitals | Medical data exchange | - Transport: HL7 and IEEE 1073  - Terminology: SNOMED-CT, ICD 10, ICD 9 and LOINC | Software: Protégé, excel, Snow Owl, Snoggle, FACT++ OWLViz, ontoGraf and HermiT  - Technology: RDF, OWL and SPARQL | Local | Semantic | RIM | The results from the queries showed that this approach were correct and clearly in alignment with its ontological commitments of supplying patient medical records in part or whole. |
| Dogac A, et al. 21011 | Turkey | The objective of this paper is to describe the techniques used in developing Turkey’s National Health Information System, a nation-wide infrastructure for sharing the EHRs. | Between CIS | Web Service  1) national health data dictionary  2) transmission data sets  3) mapping the transmission data sets to HL7 CDA schema  4) health coding reference server  5) healthcare professional registry  6) communication infrastructure (cloud)  7) CISs | Hospitals | Data sharing | - Content: ISO/TR 20514:2005, CDA and CEN/ISO 13606  - Transport: HL7  - Terminology: SNOMED-CT, ICD 10, Mesh, READ Codes and LOINC | - Technology: XML and SSL | National | Structural | RIM | There are a number of factors such as adoption of the standards, use of Core Components Technical Specification and comprehensive testing that affected the successful implementation of this approach. |
